# Creativity in design education: an educational psychology model of motivation, cognitive load, and self-efficacy

**DOI:** 10.3389/fpsyg.2026.1921917

**Published:** 2026-10-09

**Authors:** Jie Wei, Lan Liu

**Affiliations:** College of Arts and Design, Ningbo University of Finance and Economics, Ningbo, Zhejiang, China

**Keywords:** cognitive load, creative self-efficacy, design creative performance, design education, educational psychology, learning motivation

## Abstract

**Objective:**

This study examines the statistical relationships among learning motivation, cognitive load, creative self-efficacy, and expert-rated creative performance among undergraduate design students.

**Methods:**

A quantitative cross-sectional study was conducted with 428 valid responses from undergraduate students majoring in design-related disciplines. Students first completed a design task, after which questionnaire data were collected and anonymized design works were evaluated by three independent experts. SPSS was used for descriptive statistics, reliability analysis, correlation analysis, regression-based path analysis, and Bootstrap tests of statistical indirect effects; AMOS was used to evaluate the measurement model.

**Results:**

Learning motivation and germane cognitive load were positively associated with creative self-efficacy. Creative self-efficacy showed the strongest positive association with design creative performance, and germane cognitive load showed a smaller positive direct association. Extraneous cognitive load was negatively correlated with creative performance at the bivariate level, but its adjusted direct path was not statistically significant; therefore, H5 was not supported. Intrinsic cognitive load was not significant. Bootstrap results indicated significant statistical indirect relationships through creative self-efficacy.

**Conclusion:**

The findings suggest that design creativity is associated with students' motivation, meaningful cognitive engagement, and creative self-beliefs. Because the questionnaire was administered after the design task, the indirect effects should be interpreted as statistical indirect relationships rather than evidence of causal or temporal mediation.

## 1 Introduction

Creativity has long been regarded as a central outcome of education, particularly in fields where learners are expected to solve open-ended problems, generate original ideas, and transform abstract concepts into concrete outcomes. In design education, creativity is not an optional ability but a core educational goal. Design students are required to identify ill-defined problems, interpret user and contextual needs, generate multiple alternatives, evaluate visual and functional solutions, and communicate ideas through tangible design works. Therefore, creativity in design education should not be understood merely as spontaneous inspiration, but as a complex learning outcome shaped by cognition, motivation, self-beliefs, and task engagement.

Creativity is commonly defined by the combination of novelty and appropriateness or effectiveness (Runco and Jaeger, 2012). This definition is especially relevant to design education because a design outcome must be both original and suitable for a given task, audience, function, or visual context. Design creativity also involves the co-evolution of problems and solutions, as students often redefine the design problem while developing possible solutions (Dorst and Cross, 2001). In this sense, design creativity differs from general creative thinking because it is expressed through visual, material, functional, and problem-solving outcomes. Previous research on design creativity has emphasized the importance of originality, usefulness, aesthetic quality, and design evaluation (Amabile, 1982; Kaufman et al., 2008; Chaudhuri and Dhar, 2024). However, many studies in design education have focused more on teaching strategies, studio practices, digital tools, or design outcomes, while the psychological mechanisms underlying students' creative development have received comparatively less systematic attention.

From an educational psychology perspective, students' creative performance in design learning is likely to be influenced by their motivation, cognitive processing, and beliefs about their own creative ability. Learning motivation determines whether students are willing to invest effort, persist through ambiguity, and revise their design ideas. Cognitive load reflects how students allocate limited mental resources when dealing with complex design tasks. Creative self-efficacy represents students' belief that they can generate novel and valuable ideas and complete creative tasks successfully. These three constructs are central to understanding why some students can transform design learning experiences into creative outcomes, whereas others may struggle despite receiving similar instructional conditions.

Learning motivation is one of the most important psychological factors in educational achievement and creative learning. According to self-determination theory, motivated learners are more likely to engage actively, sustain effort, and experience a stronger sense of autonomy and competence (Ryan and Deci, 2000). In higher education, motivation has also been widely measured through instruments such as the Motivated Strategies for Learning Questionnaire, which emphasizes students' value beliefs, expectancy beliefs, and affective responses in learning contexts (Pintrich et al., 1991, 1993). In design education, motivation may be particularly important because design tasks are often open-ended, uncertain, and iterative. Students need to search for inspiration, generate alternatives, receive feedback, and repeatedly improve their work. Without sufficient motivation, students may avoid risk-taking, produce conventional solutions, or stop revising their ideas too early. Recent studies in educational psychology have also shown that motivation is closely related to creative thinking and creative learning outcomes (He and Chiang, 2024). Therefore, learning motivation may serve as an important antecedent of creative self-efficacy and design creativity.

Cognitive load theory provides another important perspective for understanding design learning. Human working memory is limited, and learning performance depends partly on how instructional tasks and learning environments manage cognitive demands (Sweller, 1988; Sweller et al., 2019). In general, cognitive load can be divided into intrinsic cognitive load, extraneous cognitive load, and germane cognitive load. Intrinsic cognitive load is related to the inherent complexity of the task; extraneous cognitive load is caused by unnecessary instructional or environmental burdens; and germane cognitive load refers to mental effort devoted to meaningful learning, schema construction, and deeper understanding (Leppink et al., 2013). Design learning naturally involves a high level of cognitive complexity because students must process visual information, conceptual requirements, technical constraints, and aesthetic judgments at the same time. However, not all cognitive load has the same effect. Excessive extraneous cognitive load, such as unclear task instructions, confusing tool operations, or irrelevant information, may reduce students' capacity for creative thinking. By contrast, germane cognitive load may encourage students to compare, organize, reflect on, and refine design ideas, thereby supporting creative development. Recent educational psychology research has also connected cognitive load with self-efficacy and learning processes, suggesting that cognitive load should be examined not merely as a burden but as a differentiated psychological condition in learning (Hartelt and Martens, 2024; Zhang, 2024; Sultanova, 2025).

Creative self-efficacy is another key construct for explaining creativity in design education. Based on Bandura's social cognitive theory, self-efficacy refers to individuals' beliefs in their capability to organize and perform actions required to achieve specific outcomes (Bandura, 1997). Creative self-efficacy extends this concept to creative tasks and reflects a learner's belief that they can produce creative ideas and solve problems in original ways (Tierney and Farmer, 2002). In design education, creative self-efficacy may be especially important because students often encounter uncertainty, criticism, revision, and comparison with others. Students with stronger creative self-efficacy are more likely to explore unconventional ideas, tolerate ambiguity, persist after failure, and improve their design solutions through feedback. Empirical studies have shown that self-efficacy is associated with creativity, creative cognition, and learning performance (Yan et al., 2022; Orakci, 2025; Çavuş et al., 2026). Recent research on art and design students also suggests that self-efficacy can be an important psychological factor in explaining creativity during the learning process (Jianhao et al., 2026).

Although motivation, cognitive load, and self-efficacy are established constructs in educational psychology, three gaps remain in design-education research. First, these constructs are often examined separately or in pairwise relationships rather than within one integrated explanatory model. Second, prior studies frequently treat cognitive load as a unitary burden, whereas open-ended design work requires a distinction between intrinsic task complexity, avoidable extraneous burden, and productive germane investment. Third, creative performance is commonly assessed through students' self-reports or general creativity measures, leaving uncertainty about whether the same psychological pattern is associated with the quality of actual design products. The novelty of the present study therefore lies not in proposing a new psychological theory, but in testing a domain-specific integration of motivation, differentiated cognitive load, and creative self-efficacy against an independent, expert-rated design-performance criterion in authentic undergraduate design tasks.

To address these gaps, the present study proposes an educational psychology model of creativity in design education. Rather than claiming a temporal causal process, the model tests whether learning motivation, differentiated cognitive load, and creative self-efficacy show a theoretically coherent pattern of statistical associations with expert-rated design creative performance. The domain specificity of design education is central to the model: design tasks are open-ended, ill-defined, and iterative, requiring students to negotiate problem framing, visual exploration, alternative generation, evaluative feedback, and repeated revision. Accordingly, learning motivation is distinguished from behavioral effort, germane cognitive load from general effort, and creative self-efficacy from generic academic expectancy. This domain-specific integration extends earlier work by linking internal educational-psychological states to an external product-based creativity criterion while explicitly acknowledging the reciprocal relationships that may exist in a cross-sectional design.

Based on the above theoretical discussion, this study proposes the following associational hypotheses:

**H1:** Learning motivation is positively associated with design students' creative self-efficacy.

**H2:** Creative self-efficacy is positively associated with design students' creative performance.

**H3:** Germane cognitive load is positively associated with design students' creative self-efficacy.

**H4:** Germane cognitive load is positively associated with design students' creative performance.

**H5:** Extraneous cognitive load is negatively associated with design students' creative performance.

**H6:** Creative self-efficacy statistically accounts for the indirect relationship between learning motivation and design creative performance.

**H7:** Creative self-efficacy statistically accounts for the indirect relationship between germane cognitive load and design creative performance.

Because the self-report measures were collected after completion of the design task, the hypotheses are interpreted as statistical relationships rather than as evidence of temporal or causal mediation.

By testing these hypotheses, this study aims to contribute to educational psychology and design education in three ways. First, it extends creativity research by explaining design creativity through motivation, cognitive load, and self-efficacy. Second, it introduces a more psychologically grounded model into design education research, shifting the focus from external teaching conditions to students' internal learning mechanisms. Third, it combines self-report psychological measures with expert assessment of design works, thereby providing a more comprehensive approach to evaluating creativity in design education. As shown in Figure 1, this study constructs an educational psychology model of creativity in design education.

**Figure 1:**
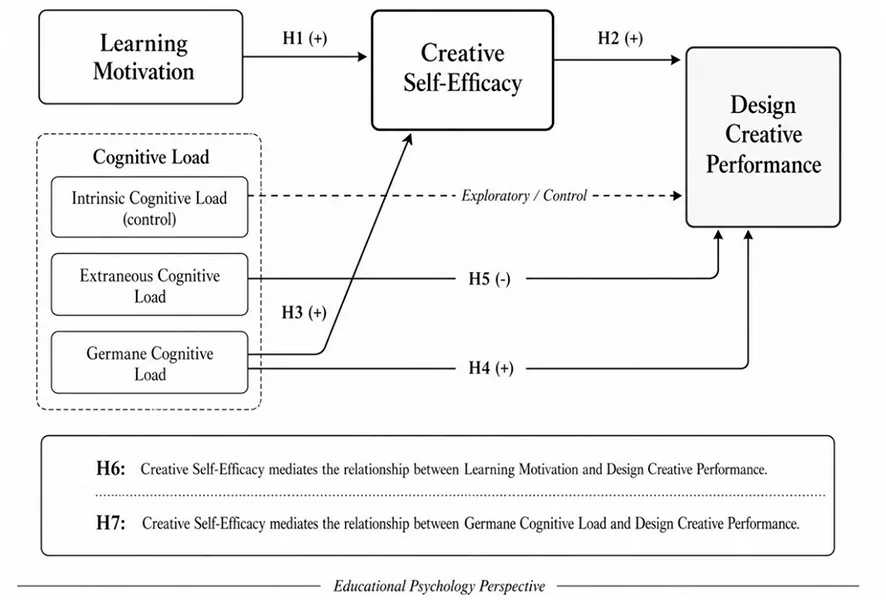
Educational psychology model of creativity in design education.

## 2 Literature review

### 2.1 Creativity in design education

Creativity is widely regarded as one of the core educational goals of design education. In general creativity research, creativity is often defined through two essential criteria: originality and effectiveness. Originality emphasizes novelty, uniqueness, and departure from conventional ideas, while effectiveness emphasizes usefulness, appropriateness, or value within a particular context (Runco and Jaeger, 2012). This dual definition is especially suitable for design education, because design works cannot be evaluated only by whether they are novel; they must also respond to practical problems, visual communication needs, functional requirements, aesthetic standards, and user expectations. Creative-thinking-oriented instruction has also been explored in vocational higher education as a means of supporting broader student development (Li et al., 2022).

Compared with creativity in general learning contexts, design creativity has stronger domain specificity. Design students usually work on open-ended tasks, such as visual communication, product design, environmental design, fashion design, or digital media design. These tasks often have no single correct answer, and students are required to generate, compare, revise, and refine multiple solutions. Dorst and Cross (2001) argued that creativity in the design process involves the co-evolution of problem and solution. In other words, designers do not simply solve a pre-given problem; rather, they gradually redefine the problem while developing possible solutions. This perspective is important for design education because students' creative performance is shaped not only by final outcomes but also by the cognitive and reflective processes through which they transform initial ideas into design works. Educational perspectives on mini-c creativity further emphasize personally meaningful creative interpretation during learning (Beghetto and Kaufman, 2007).

In design education, creativity is usually reflected in concrete works. Therefore, the evaluation of design creativity should include both psychological measurement and product-based assessment. Amabile's consensual assessment technique emphasizes that creative products can be evaluated by experts in the relevant domain (Amabile, 1982). This method has been widely used in creativity research and is particularly suitable for art and design fields, where creativity is embodied in visual, material, or functional outcomes. Kaufman et al. (2008) also pointed out that creativity assessment should consider domain characteristics and avoid relying only on general creativity tests. More recent research on design creativity evaluation further suggests that design creativity involves multiple dimensions, such as originality, usefulness, aesthetic quality, elaboration, and problem-solving value (Chaudhuri and Dhar, 2024).

Existing studies have shown that creative thinking is a central component of art and design education. Samaniego et al. (2024), through a systematic review, found that creative thinking in art and design education is associated with cognitive flexibility, originality, visual exploration, problem solving, and reflective learning. However, current design education research still tends to emphasize teaching methods, design thinking models, digital technologies, or studio-based practices, while the internal educational psychological mechanisms of design creativity remain insufficiently explained. Therefore, it is necessary to examine design creativity not only as a final learning outcome but also as a result of students' motivation, cognitive processing, and creative self-beliefs.

### 2.2 Learning motivation in design learning

Learning motivation is an important psychological factor influencing students' engagement, persistence, and learning outcomes. Self-determination theory proposes that learners are more likely to engage actively and persistently when their needs for autonomy, competence, and relatedness are supported (Ryan and Deci, 2000). In design education, autonomy is particularly important because design tasks often require students to make independent decisions about themes, forms, visual styles, materials, and solution strategies. When students perceive design learning as meaningful and self-directed, they are more likely to invest effort and persist in creative exploration.

The Motivated Strategies for Learning Questionnaire developed by Pintrich and colleagues provides an important framework for measuring students' learning motivation in higher education. The questionnaire includes motivational components such as intrinsic goal orientation, extrinsic goal orientation, task value, control beliefs, self-efficacy, and test anxiety (Pintrich et al., 1991, 1993). Although this framework was not originally developed for design education, it is highly relevant to design learning because design students' creative performance depends strongly on their perceived task value, confidence, and willingness to engage in challenging learning activities.

In design learning, motivation may influence creativity in several ways. First, motivated students are more willing to explore diverse sources of inspiration and generate multiple design alternatives. Second, they are more likely to tolerate uncertainty and ambiguity during the design process. Third, they tend to revise their works more actively after receiving feedback. Fourth, they may show stronger persistence when facing creative blocks or technical difficulties. These characteristics are important because creative design is rarely produced through a single attempt; it usually emerges through repeated exploration, reflection, and modification.

Recent educational psychology research also supports the relationship between motivation and creativity. He and Chiang (2024) found that creativity motivation plays a mediating role between creative mindsets and creative thinking. This finding suggests that students' beliefs about creativity may influence creative outcomes through motivational mechanisms. In the context of design education, students with higher learning motivation may be more likely to develop creative confidence and transform learning engagement into creative performance. Therefore, learning motivation can be understood as a psychological antecedent of creative self-efficacy and design creativity.

### 2.3 Cognitive load in design education

Cognitive load theory provides a useful framework for understanding students' learning processes in complex educational tasks. Sweller (1988) proposed that human working memory is limited, and learning may be hindered when instructional tasks impose excessive cognitive demands. Later research further developed cognitive load theory by explaining how instructional design should reduce unnecessary cognitive burden and support schema construction in long-term memory (Sweller et al., 2019).

Cognitive load is generally divided into three types: intrinsic cognitive load, extraneous cognitive load, and germane cognitive load. Intrinsic cognitive load is related to the inherent complexity of the learning task. Extraneous cognitive load is caused by unnecessary instructional design, confusing information, poor organization, or irrelevant learning demands. Germane cognitive load refers to the mental effort devoted to meaningful learning, schema construction, and deep processing (Leppink et al., 2013). This classification is important for design education because design tasks are naturally complex and cannot simply be made “easy.” The key issue is whether students' cognitive resources are consumed by irrelevant burdens or invested in meaningful design thinking.

Design learning often involves high intrinsic cognitive load. Students need to understand design problems, analyze user needs, organize visual information, master technical tools, consider aesthetic principles, and produce complete design works. Such complexity is not necessarily harmful. For design students, appropriately challenging tasks may stimulate deeper thinking and creative exploration. Therefore, intrinsic cognitive load should not always be interpreted as a negative factor.

Extraneous cognitive load, however, may have a negative effect on design creativity. If students are distracted by unclear task instructions, excessive unrelated information, complex tool operations, or poorly structured feedback, their limited cognitive resources may be diverted away from creative thinking. In this situation, students may have less mental capacity for conceptual exploration, visual experimentation, and reflective revision. Recent studies have also suggested that cognitive load is closely related to students' self-efficacy and learning experience (Hartelt and Martens, 2024). This implies that managing cognitive load is not only an instructional design issue but also an educational psychological issue.

Germane cognitive load may play a more positive role in design learning. When students actively compare design alternatives, integrate prior knowledge with new tasks, reflect on design problems, and optimize their solutions, they are devoting cognitive resources to meaningful learning. This type of cognitive effort may promote deeper understanding and stronger creative development. Therefore, this study treats germane cognitive load as a potentially positive psychological condition that supports creative self-efficacy and design creativity.

### 2.4 Creative self-efficacy and creative performance

Self-efficacy refers to individuals' beliefs about their capability to organize and execute actions required to achieve specific goals (Bandura, 1997). In educational contexts, self-efficacy influences students' learning choices, effort, persistence, emotional responses, and achievement. Creative self-efficacy extends this concept to creative tasks and refers to an individual's belief in their ability to produce creative outcomes (Tierney and Farmer, 2002).

Creative self-efficacy is especially important in design education. Design students often face open-ended problems, uncertain evaluation criteria, repeated revisions, peer comparison, and teacher critique. In such contexts, students who believe they can generate creative ideas are more likely to take creative risks, persist in difficult tasks, and continue refining their works. In contrast, students with low creative self-efficacy may avoid experimentation, rely on familiar solutions, or give up easily when their initial ideas are criticized.

Empirical research has demonstrated that creative self-efficacy is related to creative performance. Tierney and Farmer (2002) found that creative self-efficacy predicted creative performance beyond general job self-efficacy. Their later longitudinal research further indicated that creative self-efficacy can develop over time and is associated with creative performance (Tierney and Farmer, 2011). In educational contexts, Yan et al. (2022) found that self-assessment mind maps could enhance students' self-efficacy in creativity and learning performance. This suggests that students' creative self-beliefs can be cultivated through appropriate learning activities.

Creative self-efficacy may also function as a psychological bridge between learning conditions and creative outcomes. Motivation alone may not directly lead to creativity unless students believe they are capable of transforming effort into creative results. Similarly, cognitive engagement may not result in creative performance if students lack confidence in their creative ability. Karwowski and Beghetto (2019) argued that creative behavior can be understood as agentic action, meaning that individuals need both creative potential and a sense of agency to act creatively. This perspective supports the idea that creative self-efficacy is a key mediator in design creativity development.

### 2.5 Toward an educational psychology model of creativity in design education

The above literature suggests that design creativity should be examined through an integrated educational psychology framework. Creativity in design education is not merely the result of talent, inspiration, or teaching methods. Rather, it is shaped by students' motivational states, cognitive resource allocation, and creative self-beliefs. Learning motivation may encourage students to engage in design tasks, sustain effort, and seek improvement. Germane cognitive load may support deep processing, reflection, and design optimization. Creative self-efficacy may help students transform motivational and cognitive resources into creative performance.

At the same time, different types of cognitive load may have different effects. Intrinsic cognitive load reflects the complexity of the design task itself and may not necessarily reduce creativity. Extraneous cognitive load, however, may interfere with creative performance by consuming cognitive resources that could otherwise be used for problem solving and visual exploration. Germane cognitive load may support creative development because it represents meaningful cognitive investment in learning and design thinking (Jia et al., 2019; Yazici and Dogan, 2024).

Therefore, this study constructs an educational psychology model of creativity in design education that integrates self-determination theory, cognitive load theory, social cognitive theory, and creativity assessment. The integration is not intended merely to combine established constructs, but to examine how these constructs operate in a domain where tasks are ill-defined and performance is embodied in concrete design works. Intrinsic cognitive load is included mainly as a contextual control because the inherent complexity of design tasks may function as challenge rather than obstruction. Motivation and germane cognitive load are modeled as antecedent correlates of creative self-efficacy because students may develop stronger creative confidence when they value the task and invest cognitive resources in meaningful design reasoning; however, the cross-sectional design also allows the possibility of reciprocal relationships, which is addressed as a limitation.

## 3 Methods

### 3.1 Research design and participants

This study adopted a quantitative research design to examine the relationships among learning motivation, cognitive load, creative self-efficacy, and design creative performance in design education. Data were collected through a structured questionnaire and expert evaluation of students'design works. The questionnaire was used to measure students'educational psychological variables, while expert assessment was used to evaluate their creative performance in design tasks.

Participants were recruited through course-based convenience sampling from intact undergraduate design classes in the College of Arts and Design, Ningbo University of Finance and Economics, during the spring semester of 2026. The participating courses were studio- or project-based courses associated with visual communication design, product design, environmental design, fashion design, digital media art, and other design-related majors. A total of 500 questionnaires were collected. Seventy-two cases were excluded according to prespecified data-quality criteria: 16 for unusually short response time, 9 for straight-line responding, 13 for random or anomalous response patterns, 19 for failed attention-check items, and 15 for excessive missingness. The final analytic sample therefore comprised 428 students (effective response rate = 85.6%).

The final sample size was considered adequate for the planned confirmatory factor analysis and regression-based path models. With 428 valid cases and 26 indicators represented in the measurement model, the study provided more than 16 observations per indicator and exceeded commonly cited minimum sample-size recommendations for stable CFA/SEM estimation (Kline, 2016). The sample also retained substantial representation across academic years, design majors, and the two recorded learning contexts, which supported covariate-adjusted sensitivity analyses.

Learning context was recorded to describe the instructional setting in which students completed the design task. Participants came from two learning contexts: an intelligent design learning environment and a conventional project-based design learning environment. The intelligent design learning environment referred to courses that provided structured digital resources, online learning materials, or technology-assisted design guidance, whereas the conventional project-based environment referred to studio-based courses organized mainly through teacher instruction, discussion, critique, and iterative design practice.

Learning context was treated as a contextual sample characteristic rather than as a focal theoretical construct. The present study focused on psychological relationships among motivation, cognitive load, creative self-efficacy, and creative performance. Nevertheless, because learning context may shape these relationships, its exclusion from the main model is acknowledged as a limitation, and future studies should use multi-group SEM or moderation analysis to examine whether the proposed paths differ across learning environments.

The study protocol was reviewed and approved by the Ethics Review Committee of the College of Arts and Design, Ningbo University of Finance and Economics (approval no. NBUFE-SAD-REC-2026-008). Participation was voluntary. Before data collection, students received information about the research purpose, study procedures, data use, anonymity, confidentiality, and their right to decline or withdraw without academic penalty. Informed consent was obtained from all participants before questionnaire completion and use of their anonymized design works for expert evaluation.

### 3.2 Procedure and measures

The research procedure consisted of three main steps. First, students completed a course-based design task defined by the brief used in their regular class. Outputs differed by discipline and included posters or visual communication schemes, product concepts, fashion patterns, environmental-design proposals, and digital media works. Because these products are not directly identical in form, standardization focused on assessment conditions rather than forcing all students to complete the same artifact: each work responded to an instructor-defined course brief appropriate to the student's course level, was submitted as a completed design outcome, and was judged relative to the requirements of its own brief using the same five-dimension expert rubric. Second, after completing the design task, students completed the questionnaire. Third, the final design works were collected, anonymized, and randomized for expert assessment. Because the questionnaire was administered after completion of the design task, temporal precedence cannot be established; all indirect-effect analyses are therefore interpreted as statistical indirect associations rather than causal mediation (Maxwell and Cole, 2007).

Task type was not coded as a fully independent variable because output format was closely nested within major and course. To reduce confounding from domain differences, design major was included in the covariate-adjusted sensitivity analysis. Residual heterogeneity in task difficulty and output type nevertheless remains a limitation and is reported as such in the Discussion.

All psychological variables were measured using a 7-point Likert scale (1 = strongly disagree, 7 = strongly agree). Source instruments were translated into Chinese and contextually adapted for undergraduate design education. For English-language source material, one bilingual member of the research team prepared the Chinese wording and another bilingual member checked semantic consistency against the source descriptions; wording discrepancies were resolved through discussion. Before formal administration, the adapted questionnaire was reviewed by colleagues with design-education and educational-psychology experience for clarity, construct relevance, and suitability for the target students. Construct scores were calculated as the arithmetic mean of their constituent items; no reverse scoring was required, and higher values indicated a higher level of the corresponding construct.

Learning motivation was represented by six items adapted from the motivational framework of the Motivated Strategies for Learning Questionnaire (Pintrich et al., 1991, 1993), with emphasis on task value, interest, persistence, and willingness to invest effort in design learning. A sample item was: “Even when a design task is difficult, I am willing to continue improving my solution.” The six items were averaged to form the learning-motivation score.

Cognitive load was measured with items adapted from the multidimensional framework of Leppink et al. (2013). Three items assessed intrinsic cognitive load (e.g., “This design task involved relatively complex conceptual understanding”), three assessed extraneous cognitive load (e.g., “Unnecessary information interfered with my understanding of the design problem”), and three assessed germane cognitive load (e.g., “I actively compared the strengths and weaknesses of different design solutions”). Items within each dimension were averaged separately so that intrinsic, extraneous, and germane cognitive load were analyzed as distinct constructs rather than as one total cognitive-load score.

Creative self-efficacy was measured with six items adapted from Tierney and Farmer's (2002) creative self-efficacy framework and reworded for design-task contexts. Items assessed confidence in generating creative ideas, approaching open-ended problems from multiple angles, developing initial ideas into complete solutions, responding to feedback, and completing creative design tasks. A sample item was: “I believe I can generate creative design ideas.” The six items were averaged to form the creative self-efficacy score.

Design creative performance was evaluated independently by experts rather than by student self-report. Five dimensions were scored on a 7-point scale: originality, appropriateness to the brief/context, aesthetic quality, expressive completeness, and problem-solving ability. For each student, scores were first averaged across the three raters within each dimension and then averaged across the five dimensions to produce the final expert-rated creative-performance score. Higher values indicated stronger creative performance.

### 3.3 Expert assessment of design works

To improve the objectivity of creativity assessment, this study adopted an expert-based evaluation approach. Three experts were invited to evaluate the anonymized student works. All experts had professional backgrounds in design education or design practice, including experience in visual communication, product/design practice, and design pedagogy. Each expert had more than five years of teaching, research, or professional evaluation experience and was selected according to disciplinary expertise, familiarity with undergraduate design assessment, and independence from the participating classes.

Before formal scoring, the experts received a unified scoring rubric and participated in a calibration session. The rubric defined five dimensions—originality, appropriateness, aesthetic quality, expressive completeness, and problem-solving ability—and provided behavioral anchors for the 7-point scale. To support comparability across posters, product concepts, fashion outputs, environmental proposals, and digital-media works, experts were instructed to evaluate each dimension relative to the stated design brief and disciplinary function rather than to compare surface form across media. Thus, originality referred to novelty within the relevant design domain, appropriateness to fulfillment of the task/usage context, aesthetic quality to discipline-appropriate visual or formal coherence, expressive completeness to clarity and development of the concept, and problem-solving ability to the effectiveness of the proposed response. A small set of sample works from different output categories was discussed during calibration to reduce rubric ambiguity; calibration scores were not included in the analysis.

All three experts rated all 428 design works independently. The order of works was randomized for each expert. The experts were blind to students' questionnaire responses, learning context, class information, and personal identity. Ratings were completed independently without discussion during the formal scoring stage. After scoring, inter-rater reliability was evaluated using a two-way random-effects intraclass correlation coefficient for absolute agreement based on average ratings, ICC(2, k). The average-measure ICC was 0.949, 95% CI [0.934, 0.961], indicating high agreement. Because the five dimensions were theoretically defined as complementary aspects of design creative performance and showed high inter-rater consistency, scores were averaged across experts and dimensions to form the final expert-rated creative performance indicator.

### 3.4 Data analysis

Data analysis followed a two-stage framework in which AMOS and SPSS served non-overlapping purposes. First, AMOS was used only for confirmatory factor analysis of the measurement model, estimated by maximum likelihood, to evaluate factor loadings, internal consistency, convergent validity, discriminant validity, and overall measurement-model fit. Second, SPSS was used for descriptive statistics, Pearson correlations, regression-based path tests using observed composite scores, covariate-adjusted sensitivity analyses, common-method-bias diagnostics, and Bootstrap estimation of statistical indirect effects. The hypothesis decisions reported below are based on the SPSS regression/path estimates; AMOS fit indices are not used as a second, competing test of the structural hypotheses.

Before model testing, the analytic dataset was screened for missing data, outliers, and distributional problems. The 428 retained cases contained no missing values on the variables used in the main analyses, so no imputation was required. Univariate skewness and kurtosis values were within ±1.00. Multivariate outliers were examined using Mahalanobis distance across the six principal composite variables; no retained case exceeded the χ^2^(6) critical value of 22.46 at *p* < 0.001. The main hypothesis tests then used composite scale scores calculated by averaging the relevant items. Because these regressions use observed composites, measurement error is not explicitly modeled in the path coefficients; the preceding CFA is used to establish that the item sets represent their intended constructs before composite-score analysis.

For indirect-effect analysis, Bootstrap procedures with 5,000 resamples were used to estimate the statistical indirect associations involving creative self-efficacy. Bias-corrected 95% confidence intervals were reported, and an indirect association was considered statistically different from zero when the interval did not include zero. Because the self-report questionnaire was completed after the design task, these estimates are not interpreted as evidence of temporal or causal mediation.

## 4 Results

### 4.1 Preliminary analysis and sample characteristics

A total of 500 questionnaires were collected. After screening for invalid responses, 428 valid cases were retained for the final analysis, yielding an effective response rate of 85.6%. The sample included 255 female students (59.6%), 155 male students (36.2%), and 18 students who selected “other/prefer not to say” (4.2%). In terms of academic year, 116 participants were first-year students (27.1%), 124 were second-year students (29.0%), 102 were third-year students (23.8%), and 86 were fourth-year students (20.1%). The mean age of the sample was 19.89 years (SD = 1.28), and the mean length of design learning experience was 25.13 months (SD = 11.27). Sample characteristics are summarized in Table 1. The distributions of the main variables are presented in Figure 2, and their distribution and mean estimates are further illustrated in Figure 3.

**Table 1:** Sample characteristics.

Variable	Category	*n*	%
Gender	Female	255	59.6
	Male	155	36.2
	Other/Prefer not to say	18	4.2
Grade	First year	116	27.1
	Second year	124	29.0
	Third year	102	23.8
	Fourth year	86	20.1
Major	Visual communication design	110	25.7
	Product design	77	18.0
	Fashion design	84	19.6
	Digital media art	81	18.9
	Environmental design	53	12.4
	Other design-related majors	23	5.4
Learning context	Intelligent design learning environment	214	50.0
	Conventional project-based design learning	214	50.0

**Figure 2:**
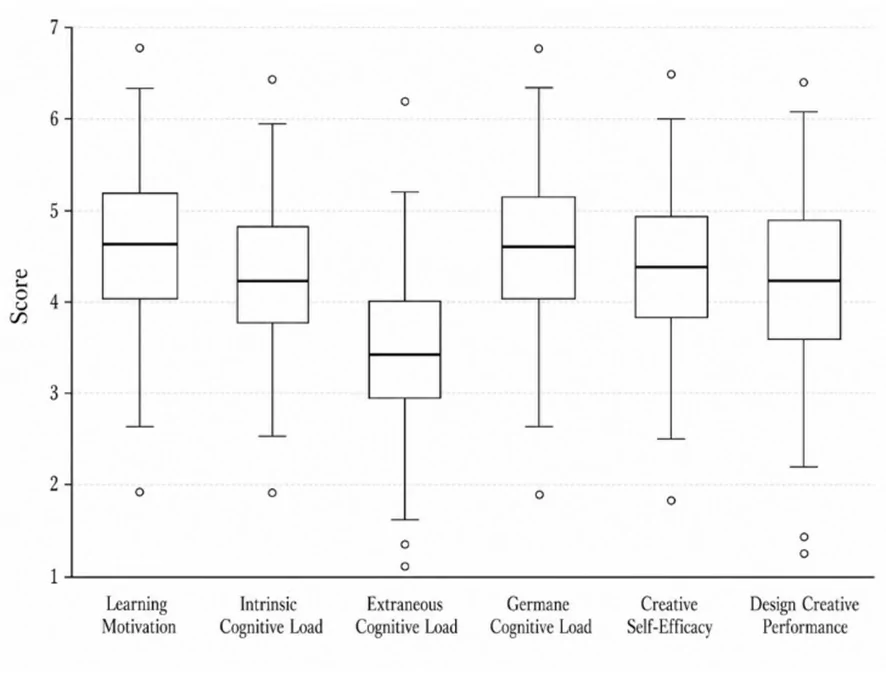
Distribution of main variables.

**Figure 3:**
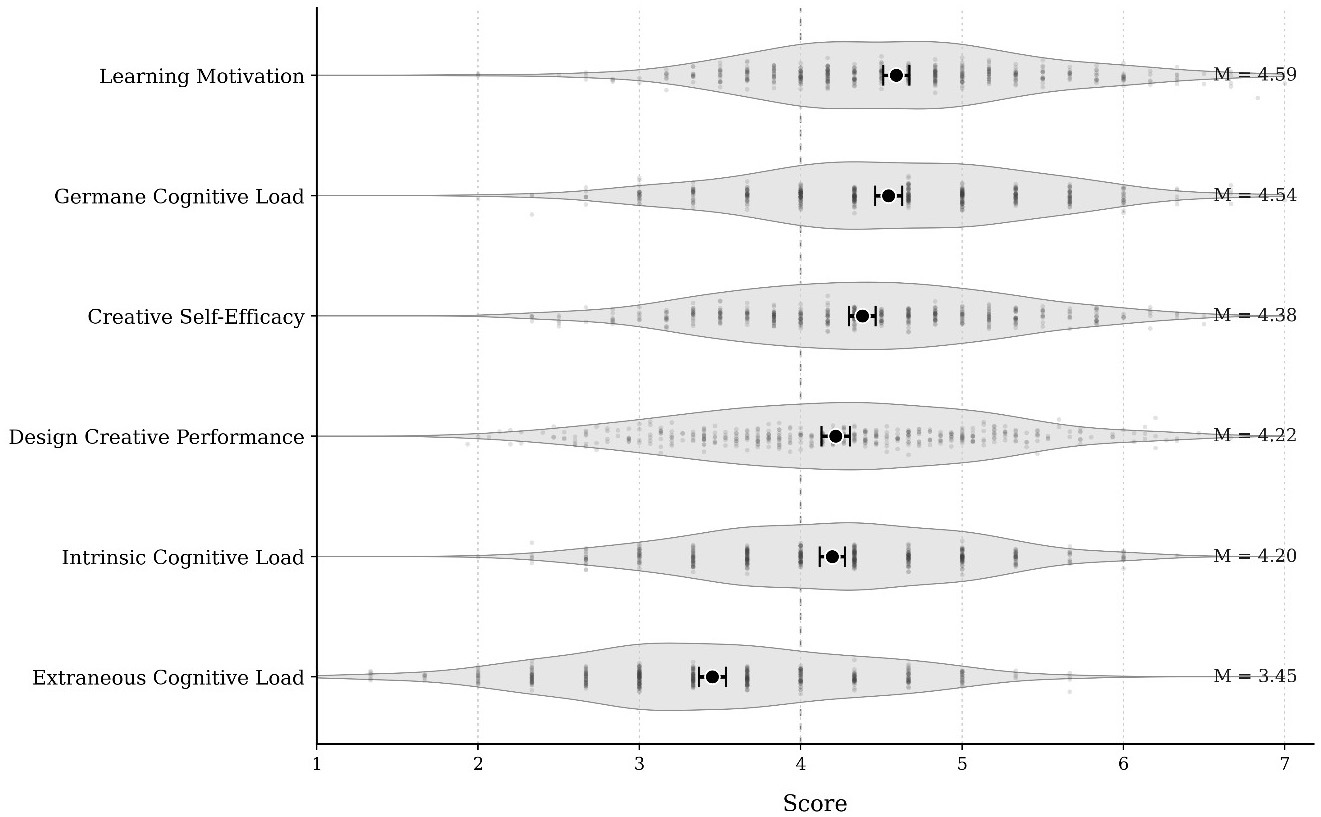
Distribution and mean estimates of the main variables.

Descriptive statistics indicated that all variables were within acceptable ranges. The absolute values of skewness and kurtosis were below 1.00, suggesting no serious deviation from normality. The mean scores of learning motivation, germane cognitive load, creative self-efficacy, and design creative performance were all above the midpoint of the 7-point scale, indicating moderate to relatively high levels across the sample. Descriptive statistics and reliability coefficients are reported in Table 2.

**Table 2:** Descriptive statistics and reliability.

Variable	M	SD	Skewness	Kurtosis	Cronbach's α
Learning motivation	4.59	0.85	0.13	0.01	0.888
Intrinsic cognitive load	4.20	0.82	–0.01	–0.44	0.742
Extraneous cognitive load	3.45	0.89	0.02	–0.35	0.801
Germane cognitive load	4.54	0.88	–0.08	–0.28	0.803
Creative self-efficacy	4.38	0.86	0.01	–0.40	0.890
Design creative performance	4.22	0.92	0.00	–0.39	0.954

The internal consistency of all scales was acceptable to high, with Cronbach's alpha values ranging from 0.742 to 0.954. These results indicate that the measurement instruments used in the study were reliable. In addition, the consistency of expert ratings was examined, and the average-measure intraclass correlation coefficient (ICC) reached 0.949, indicating a high degree of inter-rater agreement. Therefore, the mean expert score was used as the final indicator of design creative performance. The distributions of the main variables are presented in Figure 2, and their distribution and mean estimates are further illustrated in Figure 3.

### 4.2 Measurement model

Before hypothesis testing, confirmatory factor analysis was conducted in AMOS using maximum-likelihood estimation. Table 3 summarizes construct-level reliability and convergent-validity information, and Table 4 reports the standardized loading of every indicator. All item loadings were positive and substantively meaningful. Cronbach's alpha and composite reliability values were acceptable to high. AVE values were examined together with factor loadings and reliability evidence rather than as a stand-alone criterion. Discriminant validity was assessed with the Fornell–Larcker criterion by comparing the square root of each construct's AVE with its correlations with the other constructs (Fornell and Larcker, 1981).

**Table 3:** Measurement model results.

Construct	Number of items	Standardized loading range	Cronbach's α	CR	AVE
Learning motivation	6	0.68–0.84	0.888	0.896	0.592
Intrinsic cognitive load	3	0.64–0.79	0.742	0.782	0.546
Extraneous cognitive load	3	0.69–0.83	0.801	0.821	0.605
Germane cognitive load	3	0.70–0.84	0.803	0.825	0.612
Creative self-efficacy	6	0.69–0.85	0.890	0.902	0.606
Design creative performance	5	0.78–0.90	0.954	0.942	0.711

**Table 4:** Complete standardized factor loadings for the measurement indicators.

Construct	Indicator	Standardized loading
Learning motivation	LM1	0.777
	LM2	0.729
	LM3	0.766
	LM4	0.778
	LM5	0.714
	LM6	0.769
Intrinsic cognitive load	ICL1	0.673
	ICL2	0.704
	ICL3	0.723
Extraneous cognitive load	ECL1	0.713
	ECL2	0.777
	ECL3	0.781
Germane cognitive load	GCL1	0.750
	GCL2	0.756
	GCL3	0.770
Creative self-efficacy	CSE1	0.758
	CSE2	0.758
	CSE3	0.775
	CSE4	0.756
	CSE5	0.741
	CSE6	0.764
Expert-rated creative performance	Originality	0.889
	Appropriateness	0.895
	Aesthetic quality	0.909
	Expressive completeness	0.895
	Problem-solving ability	0.902

Note. Item-level loadings are reported to make the measurement model fully transparent. Expert-performance indicators refer to the five dimension scores averaged across the three independent raters before factor evaluation.

The measurement model showed acceptable fit (Table 5): χ^2^/df was below 3.00, CFI and TLI were above 0.90, and RMSEA and SRMR were below 0.08. No separate AMOS structural-model fit is used for hypothesis testing in this revision, because the substantive paths are evaluated in the regression-based framework described in Section 3.4. This separation avoids treating AMOS and SPSS as competing structural analyses.

**Table 5:** Model fit indices of the measurement model.

Model	*χ^2^*	df	*χ^2^*/df	CFI	TLI	RMSEA	SRMR
Measurement model	489.37	284	1.72	0.948	0.941	0.041	0.046

For common-method-bias assessment, Harman's single-factor test was retained only as a limited diagnostic. The first unrotated factor explained approximately 38.4% of the self-report variance and did not account for a majority of the variance. However, this result does not demonstrate the absence of common-method bias. Because learning motivation, cognitive load, and creative self-efficacy were all self-reported, residual method variance remains possible; the independently expert-rated performance outcome reduces, but does not eliminate, this concern (Podsakoff et al., 2003).

### 4.3 Correlation analysis

Pearson correlation analysis was conducted to examine the relationships among the core variables. As shown in Table 6, learning motivation was significantly and positively associated with creative self-efficacy (*r* = 0.526, *p* < 0.001) and design creative performance (*r* = 0.475, *p* < 0.001). Germane cognitive load was also significantly and positively related to creative self-efficacy (*r* = 0.416, *p* < 0.001) and design creative performance (*r* = 0.400, *p* < 0.001). Among all variables, creative self-efficacy showed the strongest positive association with design creative performance (*r* = 0.599, *p* < 0.001). Extraneous cognitive load was negatively correlated with design creative performance (*r* = –0.169, *p* < 0.001), indicating a statistically significant but relatively weak negative relationship. Intrinsic cognitive load was not significantly related to design creative performance (*r* = 0.017, *p* = 0.723). The correlation pattern among the key variables is visualized in Figure 4. The positive relationship between creative self-efficacy and design creative performance is further illustrated in Figure 5.

**Table 6:** Correlations and Fornell–Larcker discriminant validity matrix.

Variable	1	2	3	4	5	6
1. Learning motivation	**0.769**	-	-	-	-	-
2. Intrinsic cognitive load	0.028	**0.739**	-	-	-	-
3. Extraneous cognitive load	–0.081	–0.014	**0.778**	-	-	-
4. Germane cognitive load	0.358^***^	0.038	–0.140^**^	**0.782**	-	-
5. Creative self-efficacy	0.526^***^	–0.007	–0.141^**^	0.416^***^	**0.778**	-
6. Design creative performance	0.475^***^	0.017	–0.169^***^	0.400^***^	0.599^***^	**0.843**

Bold diagonal values are the square roots of AVE; below-diagonal values are Pearson correlations. In every case, the square root of AVE exceeded the absolute correlation with the other constructs, supporting discriminant validity according to the Fornell–Larcker criterion.

** *p* < 0.01.

*** *p* < 0.001.

**Figure 4:**
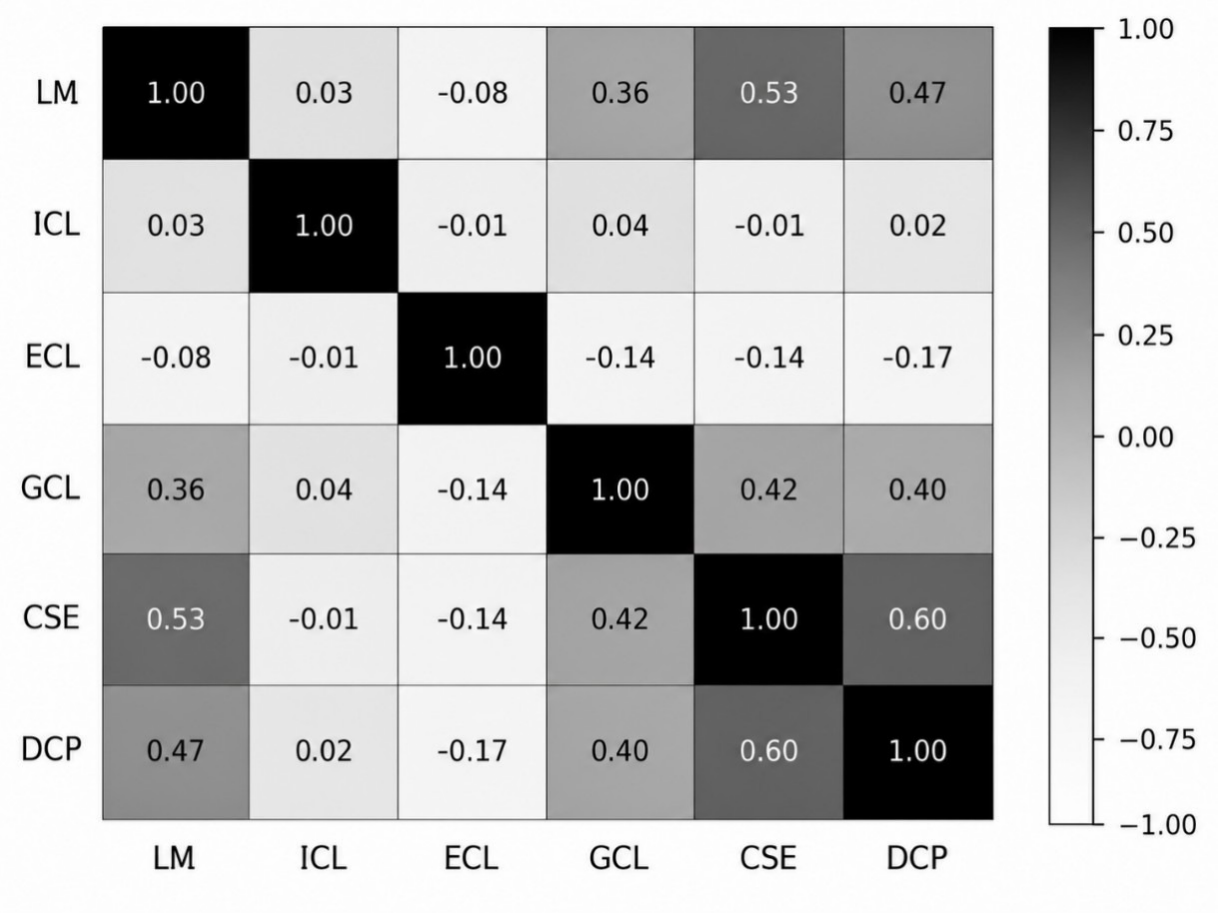
Correlation matrix of key variables.

**Figure 5:**
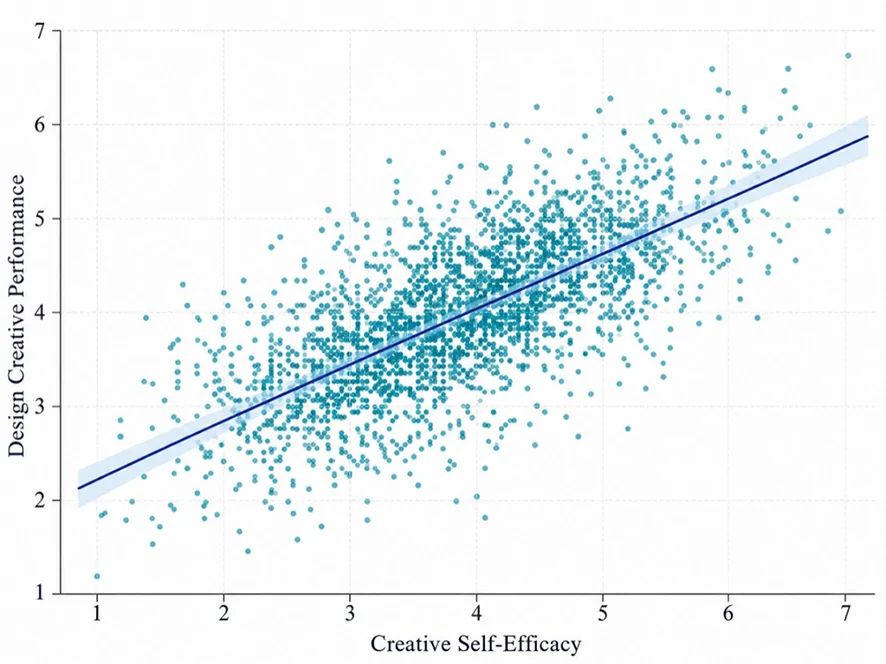
Relationship between creative self-efficacy and design creative performance.

These correlations provide preliminary support for the proposed model. In particular, the pattern suggests that learning motivation, germane cognitive load, and creative self-efficacy are positively intertwined, whereas extraneous cognitive load tends to work in the opposite direction.

### 4.4 Path analysis and hypothesis testing

To test the proposed model, a regression-based path analysis was conducted using composite scale scores. First, creative self-efficacy was regressed on learning motivation and germane cognitive load. The model was significant, *R*^2^ = 0.336, *F* = 107.50, *p* < 0.001. Learning motivation was positively associated with creative self-efficacy (β = 0.432, *p* < 0.001), supporting H1. Germane cognitive load was also positively associated with creative self-efficacy (β = 0.262, *p* < 0.001), supporting H3. The standardized path model of creativity in design education is presented in Figure 6.

**Figure 6:**
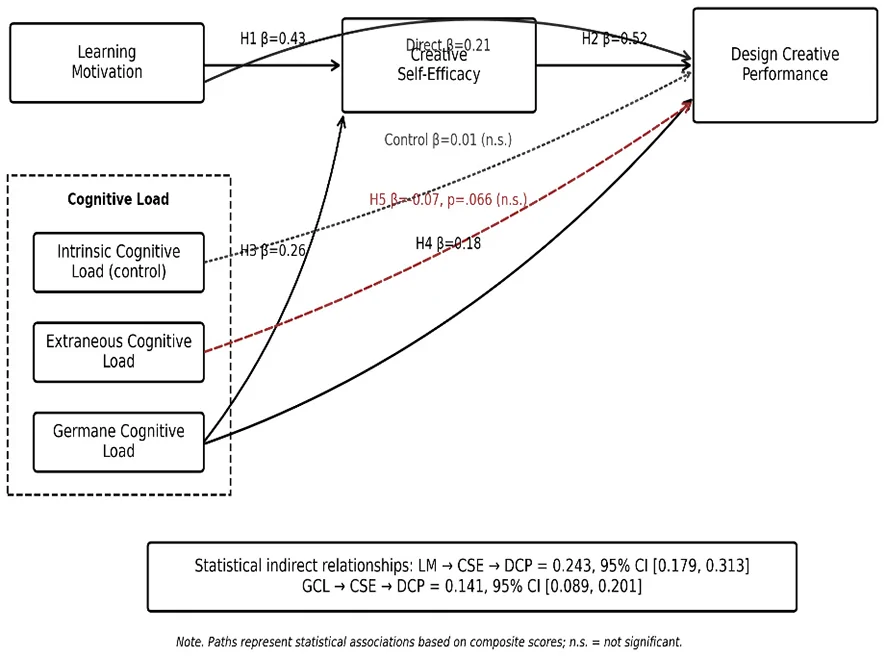
Standardized path model of creativity in design education.

Second, expert-rated design creative performance was regressed on creative self-efficacy, learning motivation, germane cognitive load, extraneous cognitive load, and intrinsic cognitive load. The direct association from learning motivation to creative performance was retained so that total, direct, and indirect associations could be estimated within the same observed-score framework. Creative self-efficacy remained the strongest positive predictor of design creative performance, and germane cognitive load showed a smaller positive association. This specification is used to describe statistical pathways only and does not assume a temporal mediation process.

Extraneous cognitive load showed a weak negative coefficient for design creative performance [β = –0.071, *p* = 0.066, 95% CI (–0.147, 0.005)]. Because this adjusted path did not reach the conventional 0.05 significance level and the confidence interval included zero, H5 was not supported. The significant zero-order correlation between extraneous cognitive load and creative performance is therefore reported only as an unadjusted bivariate association. Intrinsic cognitive load did not significantly predict design creative performance (β = 0.013, *p* = 0.730). Path coefficients are reported in Table 7.

**Table 7:** Path coefficients of the proposed model.

Path	β	SE	*t*	*p*	95% CI
Learning motivation → Creative self-efficacy	0.432	0.043	10.206	< 0.001	[0.349, 0.515]
Germane cognitive load → Creative self-efficacy	0.262	0.041	6.178	< 0.001	[0.181, 0.343]
Creative self-efficacy → Design creative performance	0.516	0.045	12.302	< 0.001	[0.428, 0.604]
Learning motivation → Design creative performance	0.214	0.046	4.716	< 0.001	[0.125, 0.303]
Germane cognitive load → Design creative performance	0.175	0.044	4.179	< 0.001	[0.092, 0.258]
Extraneous cognitive load → Design creative performance	–0.071	0.040	–1.842	0.066	[–0.147, 0.005]
Intrinsic cognitive load → Design creative performance	0.013	0.043	0.345	0.730	[–0.061, 0.087]

Overall, the path analysis suggests that creative self-efficacy was the strongest adjusted predictor of design creative performance. Germane cognitive load and learning motivation also showed positive adjusted associations, whereas extraneous cognitive load did not show a statistically significant unique effect after other variables were controlled.

A covariate-adjusted sensitivity analysis was conducted to address potential differences associated with gender, academic year, design major, design-learning experience (months), and learning context. In the model predicting creative self-efficacy, learning motivation (β = 0.433, *p* < 0.001) and germane cognitive load (β = 0.262, *p* < 0.001) remained significant after adjustment (*R*^2^ = 0.357). In the model predicting expert-rated creative performance, creative self-efficacy (β = 0.430, *p* < 0.001), learning motivation (β = 0.199, *p* < 0.001), and germane cognitive load (β = 0.135, *p* = 0.001) remained positively associated with performance; extraneous cognitive load remained small and non-significant (β = –0.073, *p* = 0.055), and intrinsic cognitive load remained non-significant (β = 0.003, *p* = 0.941; *R*^2^ = 0.445). Thus, the central pattern of findings was robust to the available demographic and learning-context controls. Task type was not independently coded and is therefore addressed as a residual limitation rather than as a separate covariate.

### 4.5 Statistical indirect-effect analysis

The statistical indirect role of creative self-efficacy was examined using 5,000 Bootstrap resamples with bias-corrected 95% confidence intervals. Because the questionnaire was completed after the design task, the following estimates are described as statistical indirect associations and do not establish temporal mediation. For learning motivation, the indirect association through creative self-efficacy was statistically different from zero. The direct association from learning motivation to expert-rated creative performance was retained, allowing total, direct, and indirect estimates to be reported without causal interpretation. The direct and indirect effects are summarized graphically in Figure 7.

**Figure 7:**
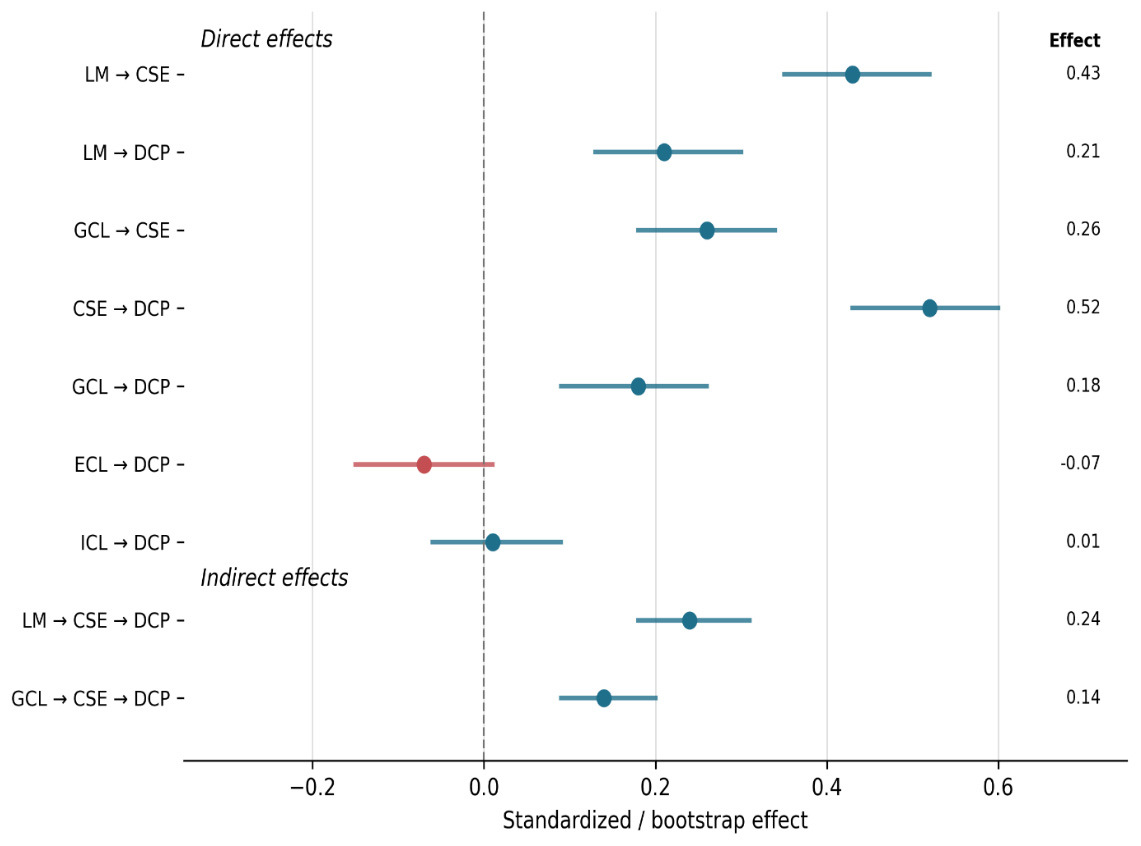
Direct and indirect effects.

For germane cognitive load, the statistical indirect association through creative self-efficacy was also different from zero, while the direct association with expert-rated creative performance remained positive. The indirect estimates are based on Bootstrap coefficients, whereas the path coefficients in Table 7 are standardized regression coefficients; therefore, the reported indirect values should not be interpreted as simple causal products of the standardized paths. Bootstrap estimates of the statistical indirect associations are reported in Table 8.

**Table 8:** Bootstrap estimates of statistical indirect associations.

Path	Total effect	Direct effect	Indirect effect (SE)	95% CI
Learning motivation → Creative self-efficacy → Design creative performance	0.457	0.214	0.243 (0.034)	[0.179, 0.313]
Germane cognitive load → Creative self-efficacy → Design creative performance	0.316	0.175	0.141 (0.029)	[0.089, 0.201]

These findings indicate that learning motivation and germane cognitive load were statistically associated with design creative performance both directly and indirectly through creative self-efficacy at the measured level. They should not be interpreted as evidence that motivation or cognitive load temporally caused changes in self-efficacy or performance in this cross-sectional design.

### 4.6 Summary of hypothesis testing

The overall hypothesis-testing results are summarized in Table 9. H1, H2, H3, H4, H6, and H7 were supported as associational hypotheses. H5 was not supported in the primary adjusted path model. H6 and H7 refer specifically to statistical indirect associations through creative self-efficacy and should not be interpreted as temporal mediation.

**Table 9:** Summary of hypothesis testing.

Hypothesis	Statement	Result
H1	Learning motivation is positively associated with creative self-efficacy.	Supported
H2	Creative self-efficacy is positively associated with design creative performance.	Supported
H3	Germane cognitive load is positively associated with creative self-efficacy.	Supported
H4	Germane cognitive load is positively associated with design creative performance.	Supported
H5	Extraneous cognitive load is negatively associated with design creative performance.	Not supported
H6	Creative self-efficacy statistically accounts for the indirect relationship between learning motivation and design creative performance.	Supported
H7	Creative self-efficacy statistically accounts for the indirect relationship between germane cognitive load and design creative performance.	Supported

In summary, the findings provide substantial empirical support for the proposed educational psychology model of creativity in design education, while also showing that not all theoretically plausible relationships were supported. Learning motivation and germane cognitive load were important correlates of creative self-efficacy, and creative self-efficacy was the strongest adjusted correlate of design creative performance. Extraneous cognitive load showed only an unadjusted negative association, and intrinsic cognitive load was not significant. These findings suggest that creativity development in design education depends more strongly on students' motivational state, meaningful cognitive engagement, and creative confidence than on task complexity alone.

## 5 Discussion

The results provide empirical support for an educational psychology model of creativity in design education, but the findings should be interpreted as statistical associations rather than causal evidence. Because students completed the questionnaire after submitting their design works, their perceptions of motivation, cognitive load, and creative self-efficacy may have been influenced by their completed design experience. This timing limits claims about psychological mediation. Therefore, the present study contributes mainly by identifying a pattern of indirect statistical relationships linking motivation, germane cognitive load, creative self-efficacy, and expert-rated creative performance in an authentic design-learning context. Recent work has also examined the relationships among generative AI, self-efficacy, and creative cognition in design students (Hwang and Wu, 2025).

The theoretical contribution of this study lies in its domain-specific integration of educational psychology constructs within design education. Motivation in this study does not simply refer to observable effort or engagement; rather, it reflects students' perceived value, interest, and willingness to persist in open-ended design learning. Similarly, germane cognitive load is not treated as ordinary effort, but as cognitive investment in meaningful problem framing, idea comparison, reflective revision, and schema construction. Creative self-efficacy is also distinct from general expectancy because it refers specifically to students' confidence in generating, evaluating, and refining creative design solutions. These distinctions are important because design tasks are less structured than many conventional learning tasks and require students to coordinate uncertainty, visual reasoning, and evaluative judgment.

The significant associations involving germane cognitive load suggest that meaningful cognitive investment may be particularly important in design education. In open-ended design tasks, cognitive challenge may support creativity when students use it to compare alternatives, reinterpret constraints, and improve design solutions. This helps explain why intrinsic cognitive load was not significant. Task complexity itself may not hinder creativity if it functions as an appropriate level of challenge. From the perspective of flow theory, challenging tasks may even support engagement when students have sufficient skills and support (Csikszentmihalyi, 1990). Compared with many structured mathematics or science tasks, in which learners typically apply more clearly specified procedures toward verifiable answers, design tasks require simultaneous problem framing, alternative generation, and evaluative revision. Accordingly, productive cognitive load in design may be expressed less as the efficient execution of a prescribed procedure and more as sustained exploration and reflective iteration (Sweller et al., 2019; Dorst and Cross, 2001). Thus, design educators should not simply reduce all forms of cognitive demand; they should reduce unnecessary burden while preserving productive challenge.

H5 was not supported in the adjusted path model. Although extraneous cognitive load was negatively correlated with design creative performance at the bivariate level, its unique effect became non-significant after creative self-efficacy, germane cognitive load, and other variables were controlled. This finding suggests that the negative role of extraneous load may be weaker or less stable than expected in design learning. One possible explanation is that design students are frequently exposed to ambiguity, critique, and iterative problem solving, and may therefore tolerate some forms of uncertainty that would be disruptive in more structured learning contexts. Another possibility is that the effect of extraneous load is partly absorbed by self-efficacy or meaningful cognitive engagement. This non-significant result is theoretically useful because it cautions against assuming that all perceived burden has an independent negative effect on design creativity.

The expert-rated outcome is a strength because it reduces reliance on self-reported creativity, but the self-reported predictors still leave room for common-method variance; Harman's single-factor result should therefore be viewed only as a diagnostic rather than proof that method bias is absent. In addition, design outputs differed across majors and task type was not independently coded. The common rubric, expert calibration, and covariate adjustment for major, academic year, design experience, and learning context reduce some of this heterogeneity but cannot remove it entirely. Future studies should use a common experimental design brief or explicitly code task type and difficulty, and should combine questionnaires with behavioral process data, design-log analysis, think-aloud protocols, eye-tracking, physiological indicators, or objective cognitive-load measures. Longitudinal or experimental designs are also needed to establish temporal ordering among motivation, cognitive load, creative self-efficacy, and performance.

Practically, the findings suggest that design educators should build learning environments that support motivation, productive cognitive engagement, and creative confidence. Specific strategies include clarifying task requirements, providing staged examples and rubrics, supporting sketching and external visualization as cognitive offloading tools, offering formative feedback during iterative revision, and using digital or AI-assisted references in ways that scaffold exploration rather than replace students' own judgment. At the same time, over-reliance on AI-generated references or ready-made solutions may narrow ideation, promote visual homogenization, and weaken students' original thinking and critical judgment. Educators should therefore require students to document their concept development, compare multiple sources, and explain how technology-assisted inputs were critically transformed rather than directly adopted. These strategies may reduce unnecessary extraneous load while preserving the productive challenge needed for creative design thinking.

## 6 Conclusion

This study examined creativity in design education from an educational psychology perspective by integrating learning motivation, cognitive load, creative self-efficacy, and expert-rated design creative performance. The results showed that learning motivation and germane cognitive load were positively associated with creative self-efficacy, and creative self-efficacy showed the strongest association with design creative performance. Germane cognitive load also showed a positive direct association with performance, whereas extraneous cognitive load was not significant in the adjusted path model and H5 was not supported.

Overall, the findings suggest that design creativity is associated with students' motivational state, meaningful cognitive engagement, and creative self-beliefs. However, because the psychological variables were measured after the design task, the indirect effects should be interpreted as statistical indirect relationships rather than causal mediation. Future research should use longitudinal or experimental designs, more objective cognitive-load indicators, and multi-group analyses across learning contexts to further clarify how educational psychology mechanisms shape creativity in design education.

## Data Availability

The raw data supporting the conclusions of this article will be made available by the authors, without undue reservation.
